# Hard-to-Heal Wound Healing: Superiority of Hydrogel EHO-85 (Containing *Olea europaea* Leaf Extract) vs. a Standard Hydrogel. A Randomized Controlled Trial

**DOI:** 10.3390/gels9120962

**Published:** 2023-12-08

**Authors:** José Verdú-Soriano, Antonio Casado-Díaz, Marisol de Cristino-Espinar, Silvia Luna-Morales, Caridad Dios-Guerra, Paloma Moreno-Moreno, Gabriel Dorado, José Manuel Quesada-Gómez, Leocadio Rodríguez-Mañas, José Luis Lázaro-Martínez

**Affiliations:** 1 Department of Community Nursing, Preventive Medicine, Public Health and History of Science, Faculty of Health Sciences, University of Alicante, 03690 Alicante, Spain; 2 Maimonides Institute of Biomedical Research of Cordoba (IMIBIC), Reina Sofía University Hospital, University of Córdoba, 14004 Córdoba, Spain; ms.cristino.sspa@juntadeandalucia.es (M.d.C.-E.); silvialu67@gmail.com (S.L.-M.); cdiosguerra@gmail.com (C.D.-G.); paloma.moreno.moreno.sspa@juntadeandalucia.es (P.M.-M.); md1qugoj@uco.es (J.M.Q.-G.); 3 Endocrinology and Nutrition Unit, Reina Sofia University Hospital, 14004 Córdoba, Spain; 4 Consortium for Biomedical Research in Frailty & Healthy Ageing (CIBERFES), Institute of Health Carlos III, 28029 Madrid, Spain; bb1dopeg@uco.es (G.D.); leocadio.rodriguez@salud.madrid.org (L.R.-M.); 5 Pharmacy Department, Reina Sofia University Hospital, 14004 Córdoba, Spain; 6 Occidente Health Center, Córdoba and Guadalquivir Health Management Area, 14005 Córdoba, Spain; 7 Department of Nursing, Faculty of Medicine and Nursing, University of Cordoba, 14004 Córdoba, Spain; 8 Department Bioquímica y Biología Molecular, Campus Rabanales C6-1-E17, Campus de Excelencia Internacional Agroalimentario (ceiA3), Universidad de Córdoba, 14071 Córdoba, Spain; 9 Department of Geriatrics, University Hospital of Getafe, 28905 Getafe, Spain; 10 Diabetic Foot Unit, University Podiatry Clinic, Complutense University of Madrid, 28040 Madrid, Spain; diabetes@ucm.es

**Keywords:** hard-to-heal wound, EHO-85, amorphous hydrogel, randomized active-controlled trial, *Olea europaea* leaf extract

## Abstract

Chronic wounds, especially those that are hard-to-heal, constitute a serious public-health problem. Although progress has been made in the development of wound dressings for healing, there is little high-quality evidence of their efficacy, with no evidence of superiority in the use of one hydrogel over another. To evaluate the superiority of a hydrogel (EHO-85), containing Olea europaea leaf extract (OELE), over a standard hydrogel (SH), the promotion and/or improvement of healing of difficult-to-heal wounds was compared in a prospective, parallel-group multicenter, randomized, observer-blinded, controlled trial (“MACAON”). Non-hospitalized patients with pressure, venous or diabetic foot-ulcers difficult-to-heal were recruited and treated with standard care, and EHO-85 (n = 35) or VariHesive (n = 34) as SH. Wound-area reduction (WAR; percentage) and healing rate (HR; mm^2^/day) were measured. EHO-85 showed a statistically significant superior effect over VariHesive. At the end of the follow-up period, the relative WAR decreased by 51.6% vs. 18.9% (*p* < 0.001), with a HR mean of 10.5 ± 5.7 vs. 1.0 ± 7.5 mm^2^/day (*p* = 0.036). EHO-85 superiority is probably based on its optimal ability to balance the ulcer bed, by modulating pH and oxidative stress. That complements the wetting and barrier functions, characteristics of conventional hydrogels. These results support the use of EHO-85 dressing, for treatment of hard-to-heal ulcers. Trial Registration AEMPS:PS/CR623/17/CE.

## 1. Introduction

Chronic wounds are a major problem for public health-systems worldwide [1]. Over a lifetime, 1–2% of the population, especially older adults, will have suffered a chronic injury of mixed etiology [2,3], and the prevalence will predictably rise, due to population ageing, and the increase in diabetes, obesity and cardiovascular disease [4]. Patients with chronic wounds, regardless of age, sex, and wound etiology, have a higher morbidity [5] and mortality risk than the general population [6]. Therefore, these injuries constitute a significant burden for patients, caregivers, public health-care systems, health-care providers, and society at large [1]. They affect quality of life, increasing consumption of social-care resources, including both direct and indirect costs [7]. Within the group of chronic wounds, there is a special subgroup of patients with so-called hard-to-heal wounds, which heal over a long period of time, have a torpid course of more than six months, and have a significantly worse prognosis in all aspects [8].

Therefore, promoting the healing process and shortening the duration of chronic wounds, especially hard-to-heal ones, are of paramount importance. That can be accomplished by designing cost-effective, easily applied dressings that provide good outcomes. Yet, that represents a significant challenge in modern clinical medicine [4,9]. Many advanced wound dressings are available, but there is little high-quality evidence from randomized clinical trials (RCTs) to support their use. Thus, recent meta-analyses and systematic reviews on the use of different dressings in the treatment of pressure and venous ulcers suggest the need for more complete and higher quality clinical trials to obtain conclusive results [10,11]. In this context, multifunctional hydrogel dressings, capable of modulating wound microenvironments, activating the healing process [12] with an appropriate cost-effectiveness ratio [13,14], are of great interest.

For this purpose, we developed EHO-85, a multifunctional hydrogel, the main component of which is *Olea europaea* leaf extract (OELE). OELE has important biological activities, including high antioxidant capacity because of its rich polyphenol content [15]. This allows that its application in cutaneous wounds, characterized by high oxidative stress, counteracts the negative effect of a high content of reactive oxygen species (ROS) and favors wound healing [16]. Thus, EHO-85 has been designed with a holistic therapeutic approach, to act on the wound bed, which has demonstrated excellent wound-healing effectiveness and ease of application [16,17]. Indeed, a multicenter, randomized, active-controlled clinical trial showed that EHO-85 significantly accelerated wound healing. That was particularly significant in the early stages of healing, independently of ulcer etiology (venous, pressure or diabetic foot), doubling the reduction in wound area, compared to a commonly-used hydrogel [18]. Therefore, the aim of the present study was to evaluate, based on the pivotal trial, the efficacy of EHO-85 hydrogel treatment in improving wound closure, in patients with chronic hard-to-heal ulcers with a torpid course, compared to a commonly used standard hydrogel dressing.

## 2. Results and Discussion

### 2.1. Description of the Study Population and Typology of Hard-to-Heal Wounds

**Figure 1:**
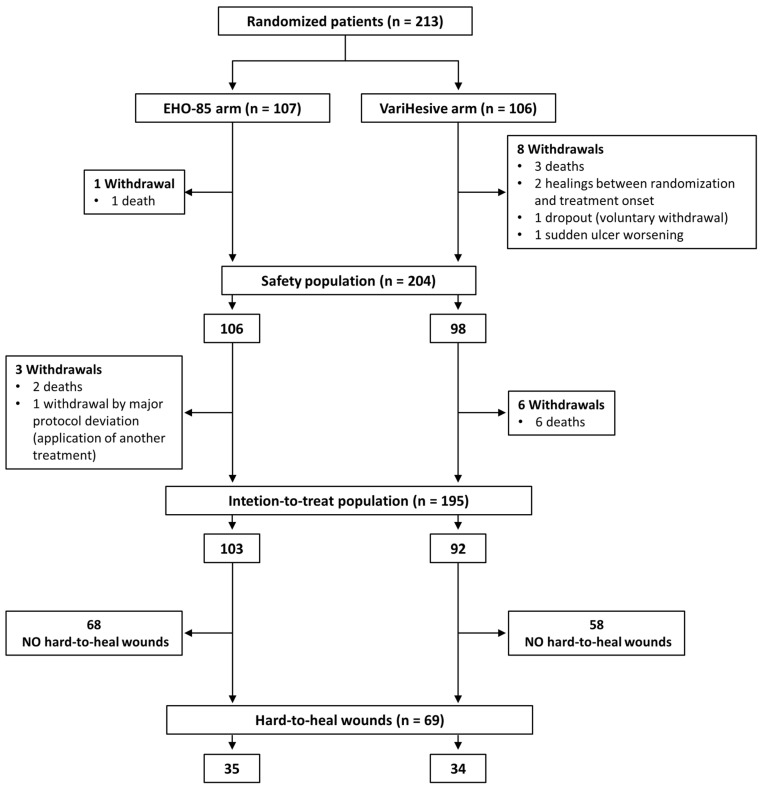
Diagram CONSORT profile.

In the pivotal clinical trial, from November 2018 to June 2019, 213 patients were enrolled and randomized to be treated with either EHO-85 (n = 107) or VariHesive (n = 106). Eighteen patients (four in the EHO-85 group and 14 in the control one) could not be included in the intention-to-treat (ITT) analysis. This was mainly due to the fact that 67% of these patients died (Figure 1). Therefore, 195 patients (92%) who had received at least one application of any of the investigational treatments, and from whom at least one post-treatment digital image had been obtained, formed the ITT analysis population (103 treated with EHO and 92 with VariHesive). Of those, 68 of the EHO-85 group and 58 of the VariHesive one were non-hard-to-heal. Thus, the evaluated population of hard-to-heal ulcers included 69 patients (35 treated with EHO-85 and 34 with VariHesive) (Figure 1).

The baseline characteristics of the patients with hard-to-heal wounds are shown in Table 1. They are broadly similar to the overall patient population included in the pivotal trial [18]. Table 2 shows the baseline ulcer characteristics of the patients with a torpid course. Sociodemographic data, ulcer characteristics and previous local treatments were balanced between the two groups at baseline (Table 1 and Table 2).

Overall, pressure ulcers (PU) were the most frequent (56.5%), followed by venous leg (VLU) (40.6%) and diabetic foot ones (DFU) (2.9%). Most ulcers were single ones (56.5%), and 32.4% being recurrent. The ulcers were on average 16.7 months old, with an area of 10 cm^2^ or less (33.3%). Granulation tissue covered an average of 76.8 ± 37.2%, and most ulcers had medium (34.8%) or low (43.5%) exudate levels. In accordance with clinical practice guidelines, most patients included in the study (81.2%) were being cared with moist wound dressings, and 50.7% needed debridement in the 30 days before the start of treatment, mainly enzymatic (44.4%) and/or autolytic. Similarly, 34.8% were treated treatment for wound infections. The vast majority of VLU (95.8%) were being treated with therapy of compression. In addition, most patients with PU were using pressure-relieving mattresses (93.8%) and following repositioning protocols (99.1%). More than half of the ulcers were recurrent (52.2%). These ulcers constitute a major social, psychological and financial burden, both for individual patients, families and health systems [19]. They also have a poorer prognosis, often requiring hospitalization [20]. In fact, ten of the 69 hard-to-heal ulcers included in the trial (14.5%) had required hospital admission at some point, eight of which were randomized to EHO-85 treatment, and the remaining two to standard hydrogel.

### 2.2. Wound-Area Reduction and Healing Rate

**Figure 2:**
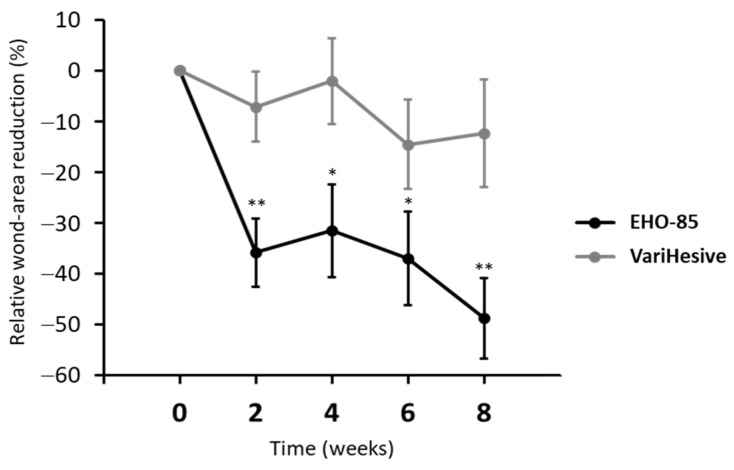
Evolution of reduction in ulcer area. Percentages in both groups of treatment during the eight-week follow-up are shown for ITT, relative to WAR evolution. Results are expressed as mean ± SD. ** *p* < 0.001 and * *p* < 0.01 for comparison of EHO-85 vs. VariHesive.

The EHO-85-treated group showed a significantly superior effect than the VariHesive-treated one on wound-area reduction (WAR) and healing rate (HR), accelerating wound healing in hard-to-heal ulcers. The results of WAR analyses at the end of the eight-week follow-up trial show that wound reductions were significantly greater in ulcers of patients treated with EHO-85. Median relative WAR dropped by 51.6% in the EHO-85-treated group, versus 18.9% in the VariHesive one (*p* = 0.008). WAR in the group treated with EHO-85 was always higher during the whole study (*p* < 0.01). Figure 2 shows the evolution of ulcer-closure reduction in each treatment group, throughout the trial. The intensity of the reduction in ulcer area observed in the group treated with EHO-85, after the first two weeks of treatment, was particularly noteworthy (*p* < 0.001). Differences continued to increase, until they reached their highest value at the end of the trial (−48.72% *vs.* −12.32%). Thus, one in three patients treated with EHO-85 achieved a closure rate of at least 80%, compared to only 9.1% among those treated with standard hydrogel. That shows the positive effect of EHO-85 on healing, considering that both groups received the same standard of care, as recommended by clinical-practice guidelines. This favorable response to EHO-85 will potentially have a major impact on the quality of life and morale of patients with hard-to heal ulcers, families and caregivers,—who see an otherwise typically slowly resolving ulcer—heal faster [20].

**Figure 3:**
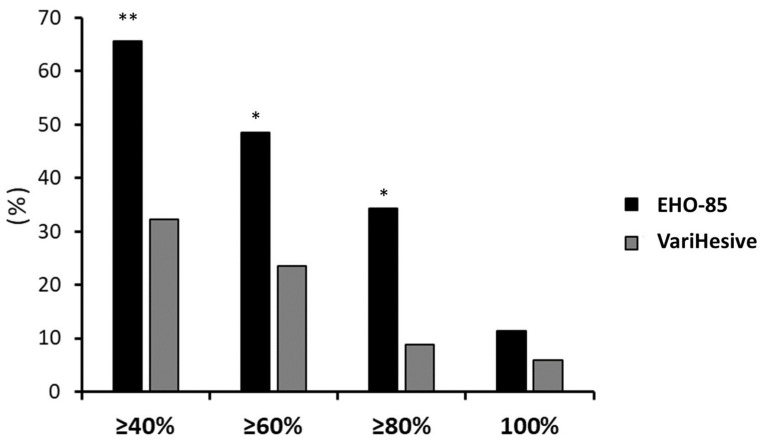
Percentages of patients with hard-to-heal wounds who achieved healing. Data are shown for ≥40%, ≥60%, ≥80% and 100% healing. Analyses by ITT. ** *p* < 0.001 and * *p* < 0.01, for the comparison of EHO-85 vs. VariHesive.

Analyses of effectiveness of the new EHO-85 treatment on ulcers, with more than six months of evolution, was completed by comparing the HR or daily reduction in ulcer area in absolute terms (mm^2^ reduction/day of treatment) between the two treatments. Hard-to-heal ulcers in patients treated with EHO-85 showed an average daily reduction of 10.5 ± 5.7 mm^2^ during the eight-week trial, compared to 1.0 ± 7.5 mm^2^ in the group treated with the comparator hydrogel (*p* = 0.036). In the EHO-85-treated group, WAR of over 40% of baseline was significantly higher (65.7 vs. 30.3 patients; *p* < 0.001). Stricter criteria (WAR ≥ 60% and ≥80%) confirmed the superiority of the EHO-85 treatment, over the positive control (*p* < 0.01; 48.6 vs. 21.2% and 34.3 vs. 9.1%, respectively). Figure 3 shows the percentage of patients with hard-to-heal wounds achieving various levels of healing (≥40%; ≥60%; ≥80%; and 100%) after eight weeks of follow-up. Despite a significant difference at the clinical level between the patients achieving complete ulcer healing in only eight weeks [four patients (11.4%) for EHO-85 vs. two patients (6.1%) for VariHesive positive control], the difference was not statistically significant.

**Figure 4:**
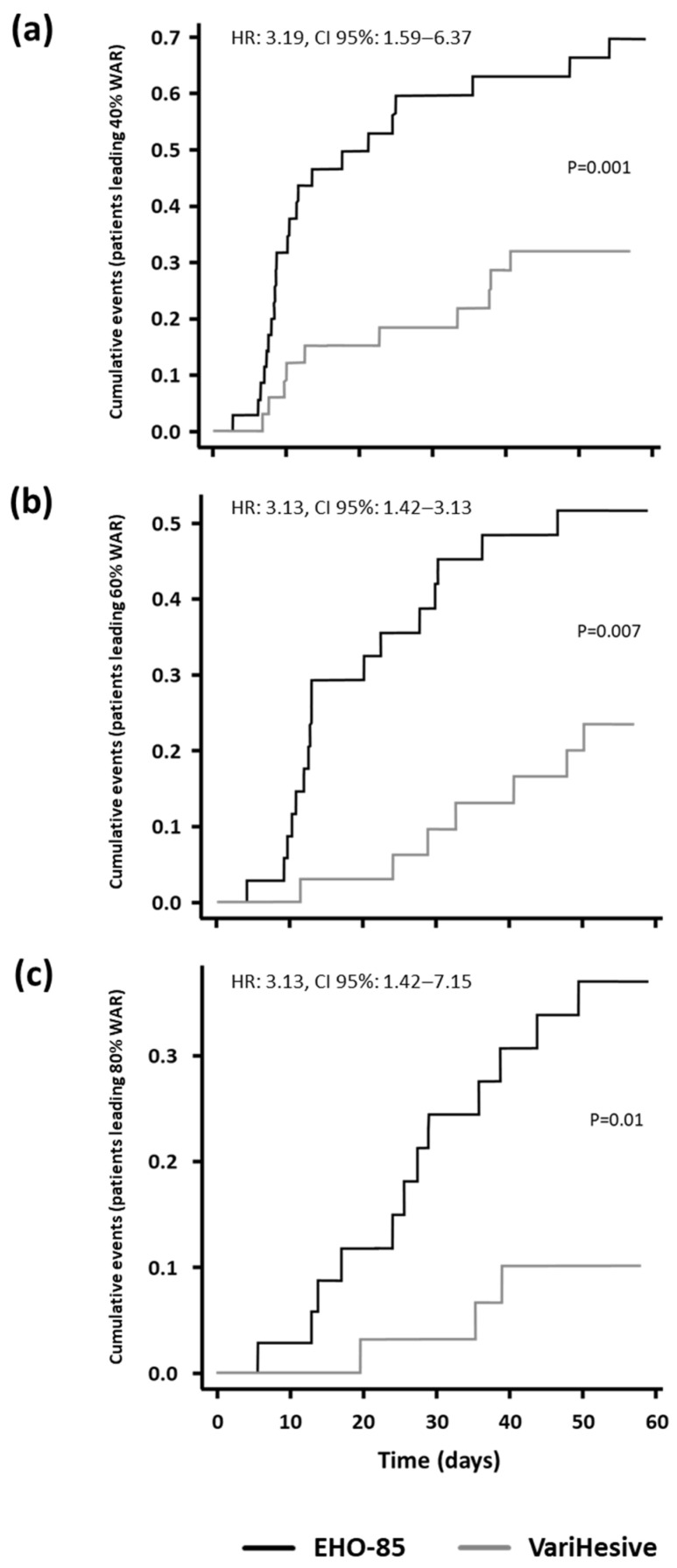
Kaplan-Meier’s curves. Cumulated incidence of patients with ulcer healing ≥40% (**a**), ≥60% (**b**) and ≥80% (**c**) for ITT.

In this regard, Kaplan-Meier analyses showed that EHO-85 hydrogel treatments were associated with a higher probability of achieving a WAR ≥ 40%, with more than three times the odds compared to the positive control (HR 3.19; CI 95%1.59–6.37). Similar values were found for WAR ≥ 60% and ≥80% (hazard ratios of 3.13 and 3.13, respectively) (Figure 4).

The capacity of the EHO-85 amorphous hydrogel to enhance the healing of torpid ulcers could probably be due to microenvironment modulation in these lesions. That could be accomplished acting on the alkaline pH [21] and on excess of ROS [22] that characterize chronic ulcers, through its acidifying and antioxidant properties [16]. These actions complement its characteristics as a wetting agent and ulcer-bed protector, inherent to a hydrogel. Indeed, they are biomaterials designed to provide three-dimensional scaffolds and support structures in wound beds. Besides, they have the ability to donate and absorb fluids without dissolving, allowing to maintain a moist environment. They also create a barrier of mechanical protection and thermal insulation, which adapts to the wound. Additionally, they allow diffusion of nutrients, metabolites and water-soluble molecules, as well as infiltration of cells, being a platform of high permeability, facilitating healing [13]. Among wound dressings, amorphous hydrogels are considered a reference. They can be used at any phase of the healing process, for any type of wound or ulcer (venous, pressure, diabetic, surgical, chemical, physical-mechanical, burn, etc.) [23].

Acidification of ulcer pH is an important contributor to healing. The pH of the skin is between 4.7 and 5.75. This creates an antimicrobial barrier because most pathogenic microorganisms that can induce infection need a pH higher than 6 to develop. Therefore, maintaining the acid pH of the skin after a wound reduces the likelihood of infection, which is one of the main causes of the chronification of skin ulcers [24]. In addition, when a wound occurs, numerous proteases of microbial and inflammatory cell origin are released, which degrade the tissue and, if maintained over time, impede tissue regeneration. The optimum pH for the activity of these proteases is generally alkaline (approximately 8), therefore lowering the pH in the wound inhibits the activity of these proteases and promotes healing [25]. EHO-85 increases acidity in the wound bed upon application, being maintained over time [17]. That should help to prevent microbial colonization [26]. Regenerative processes such as angiogenesis, metalloproteinase functionality and macrophage and fibroblast activation are also induced in an acidic environment [27]. Besides, due to the Bohr-effect, the acid pH favors a greater availability of oxygen to the cells in the ulcer. This facilitates healing. Thus, when the partial pressure of O2 (PO2) is greater than 40 mm Hg, wound healing is greater in chronic ulcers than at PO2 < 20 mm Hg [28,29]. Therefore, an acid pH in the ulcer bed, such as that induced by EHO-85, can significantly contribute to healing [27].

In the microenvironment of chronic wounds, such as hard-to-heal ones, there is a high level of ROS [22], resulting in degradation and deterioration of cellular components and functions. That may contribute to a sustained and uncontrolled inflammation process [30], leading to slowing down, and even stopping, repair processes of ulcerated tissue at all stages [31]. Therefore, reduction of ROS, through the use of dressings containing natural antioxidant free-radical scavengers, can be an appropriate strategy to accelerate wound healing [32,33], especially for hard-to-heal ulcers. The antioxidant capacity of flavonoids, oleuropeosides, and polyphenols contained in OELE, main component of eho-85, has been widely evaluated [16,34]. Among the polyphenols, the most abundant compound is oleuropein, followed by hydroxytyrosol, oleuropein aglycone and tyrosol. Besides, their antioxidant activity is synergistic [35]. The treatments the wounds with oleuropein or OELE significantly accelerated healing of cutaneous ulcers, through its antioxidant properties [36].

The EHO-85 hydrogel can accelerate closure of difficult-to-heal wounds, due to the wound microenvironments modulation through its multifunctional properties [16,17]. Nevertheless, to the present, the most recent available guidelines and systematic reviews have not provided conclusive evidence of differences in efficacy between hydrogel dressings, in relation to healing of ulcers. That includes pressure [37], diabetic foot or venous ones [11]. In fact, evidence supporting the adoption of a particular intervention in ulcer management is sparse, limited and even inconsistent [38].

The results of the present study have clinical relevance, with strength and importance in relation to the state of the art, providing strong evidence of superiority of one hydrogel over another. That stems from the prospective, parallel-group, randomized, multicenter, investigator-blinded, prospective, parallel-group trial used. Thus, EHO-85 dressing improved the rate of wound closure in eight weeks, in patients with hard-to-heal wounds, compared to a conventional standard hydrogel widely used for decades in routine wound-healing practice worldwide. This confirms the concept that modification of the wound microenvironment, together with the best treatment standards according to the etiology of the ulcer, can promote and/or accelerate the healing process of skin ulcers, including chronic hard-to-heal ones, even at a very early stage [39,40].

Besides, the rheological characteristics of the EHO-85 amorphous hydrogel make it easy to apply to wounds by gentle manual pressure, facilitating deposition on wounds with simple manipulation, compared to other usual hydrogels, which could facilitate better compliance in wound care [41].

The main limitation of the present clinical trial could be the eight-week follow-up period, which might be too short to detect significant differences in the extent of complete healing [42]. In any case, such period is widely accepted and increasingly used by the scientific community. In fact, it is considered a valid surrogate marker for predicting healing of chronic ulcers [43,44,45]. Indeed, its use in clinical trials on skin ulcers is expanding, precisely because of the long time required for these lesions to reach complete healing. Furthermore, its validity is reaffirmed by results of clinical trials conducted in recent years, evaluating the efficacy of products for treatment of skin ulcers [40,42,46]. Even shorter time periods (four and six weeks) have been proposed as valid markers, to demonstrate the potential clinical efficacy of products for ulcer treatment [44,45,47,48].

## 3. Conclusions

In conclusion, based on the evidence presented, the EHO-85 amorphous hydrogel could be proposed in evidence-based clinical practice guidelines as a recommendation for treatment of hard-to-heal ulcers, with a strong recommendation grade (A) [49].

## 4. Materials and Methods

### 4.1. Study Design

This study represents a secondary objective of a prospective, parallel-group, randomized, multicenter, investigator-blinded, prospective trial, approved by the Ethics Committee of “Reina Sofía” University Hospital of Cordoba (Spain) and by the Spanish Agency for Medicines and Health Products (AEMPS; PS/CR 623/17/EC, dated 18 August 2018), designed to evaluate the performance of EHO-85 hydrogel versus a standard hydrogel, commonly-used one in the treatment of hard-to-heal ulcers.

EHO-85 is an amorphous hydrogel designed and evaluated by our group [16,41], made of (1) purified water; (2) triethanolamine (TEA), an agent for gelling and hydrogel network formation [50]; (3) carbopol 980, a crosslinked acrylic-acid polymer with very good rheological properties, easily and rapidly dispersible, providing an effective insulating barrier to wound beds [50]; (4) Olea europaea leaf extract (OELE), the active compound, included for its antioxidant properties in the wound bed [16]; (5) disodium salt of ethylenediaminetetraacetic acid (Na_2_-EDTA), for its antimicrobial and antibiofilm properties [51]; (6) geogard ultra (gluconolactone, sodium benzoate and calcium gluconate) as an antimicrobial; (7) glycerin [52]; and (8) fucocert (L-fucose, D-galactose and galacturonic acid) [53], moisturizing and self-emulsifying agents, which create a film on the ulcer for its protection, being important for elasticity and repair. The formulation also includes an acid pH (range 5.0–5.5). All this provides to EHO-85 hydrogel suitable properties to create a wound environment that promotes and facilitates wound healing [27].

The positive control, comparator product, was VariHesive hydrogel; marketed in various countries around the world under other trade names, such as DuoDERM Hydractive Gel, DuoDERM Hydroactive Sterile Gel, DuoDERM Gel Hidroactivo and GranuGEL. It is composed of sodium salt of carboxymethylcellulose, propylene glycol, pectin and water. It was applied according to the manufacturer’s instructions. The clinical trial involved nurses specialized in ulcer treatment from 24 Health Centers (see Supplementary Material Table S1), who were trained to unify care protocols and attended a course on good clinical practice at the Andalusian School of Public Health (Granada, Spain).

### 4.2. Participants and Procedures

Patients of both sexes, older than 18 years, diagnosed of VLU, DFU of neuropathic origin, grade I or II according to the Wagner scale were recruited [54], or PU of category II or III, according to the European Pressure Ulcer Advisory Panel (EPUAP) [55]. When there was more than one ulcer, the one that best matched the selection criteria was selected. Exclusion criteria were strictly defined (detailed in Table S2 of Supplementary Material). In addition, specific exclusion criteria were defined for each ulcer type (Table S3). In case that wound debridement and/or infection control was necessary, initiation of therapy was postponed from 24 to 72 h, after completion of debridement. All subjects gave their informed consent for inclusion before they participated in the study. The study was conducted in accordance with the Declaration of Helsinki, and the protocol was approved by the Ethics Committee of “Reina Sofía” University Hospital of Cordoba (Spain) and by the Spanish Agency for Medicines and Health Products (nº AEMPS; PS/CR 623/17/EC).

The target wound was cleaned using 0–9% sodium chloride solution, and further treated with EHO-85 hydrogel (n = 35) or VariHesive from ConvaTec (Reading, UK) as control standard hydrogel (CSH; n = 34; hydrogel characteristics described in Supplementary Material), three times/week, every other day, for eight weeks, or until complete healing, with a maximum of 24 applications. Subsequently, Mepilex silicone-foam dressing from Molnlycke Health Care (Gothenburg, Sweden) was applied. Both groups received the same standard care, according to clinical practice guidelines. No other general or local treatment was applied. In those cases in which dressing changes were greater than three per week, due to the amount of exudate or the clinical evolution of the wound, the same treatment protocol was applied for the additional wound care visits, except for the treatment with the studied hydrogel or CSH. In case of wound infection, the frequency of cleansing and secondary dressing changes was enhance. Moreover, Acticoat/Argencoat nanocrystalline silver dressing from Smith & Nephew (Watford, UK) was applied, until remission of the infection.

Patients with PU complied with a standardized repositioning regimen. In patients with VLU, compression therapy was mandatory. Thus, Indacrep elastic-compression bandage from Laboratories Indas—Attindas Hygiene Partners (Raleigh, NC, USA) was applied over the secondary dressing every 24 or 48 h, according to the needs of each patient. Likewise, patients with DFU were treated with felted foam, in combination with appropriate footwear.

Wound response to treatment was assessed by digital photographs taken at the first visit, start of therapy, and each fortnightly visit, until week eight. If the wound healing was complete, or there was a patient withdrawal or discontinuation of treatment, the last visit would be performed earlier. A photograph was taken at that time, to document the status of the wound. Two eight-megapixel photographs of the wound were taken and sent for evaluation by an experienced, software-trained investigator, who had not been involved in treatment delivery, being unaware of the type of dressing used. The wound images were labelled with a standard description including patient code, date, and a millimeter ruler, for both granulation tissue-area measurements and digital wound planimetry, using PictZar Pro version 7.5.1 from Advanced Planimetric Services (Elmwood Park, NJ, USA).

### 4.3. Randomization and Stratification

After written-informed consent to participate in the trial was recorded from the patients or their legal representatives, demographic characteristics, medical-surgical and ulcer history of patients were documented at the screening visit. Etiology, location, treatment during last month, duration and size of assessed ulcers were recorded on the forms. Investigators confirmed that all eligibility criteria and none of the exclusion criteria were met for each patient.

An electronic data collection form, based on Research Electronic Data Capture (REDCap) software version 7.06 from Vanderbilt University (Nashville, TN, USA), was used at the Data Processing Centre of the University of Córdoba (Córdoba, Spain). Data were anonymized and appropriate measures were adopted to ensure patient confidentiality.

The study’s internal validity was reinforced by a randomized, stratified, assessor-blinded design. In this way, selection and confounding biases are minimized, so that treatment is the only relevant difference between the two evaluated groups.

Stratified randomization was performed using REDCap programmed for this purpose by the Innovation Department of the “Instituto Maimónides de Investigación Biomédica de Córdoba” (IMIBIC, Córdoba, Spain), of the Andalusian Public Health System (Spain). At a first level, stratification respect the etiology of the ulcer (PU, VLU or DFU) and subsequently the area (cut-off point of 10 cm^2^) and the duration (cut-off point of six months) of the ulcer [56,57].

### 4.4. Outcomes

The main outcome of this study was the percentage of the relative reduction in WAR, calculated as [(A_t_ − A_0_)/A_0_] × 100. Being A_t_ the last (day) area (mm^2^) measurement obtained, and A_0_ the first one at time zero. Absolute WAR corresponds to At − A0. Daily wound HR was calculated as [(A_t_ − A_0_)/t] (mm^2^ per day of treatment). Percentage of patients with a relative WAR ≥ 40%, ≥60%, ≥80% and 100%were calculated at final available measurement.

### 4.5. Statistical Analyses

Statistical analyses were performed at the IMIBIC Innovation Department, by an independent third party, using R software version 4.0.3 from R Foundation for Statistical Computing (Vienna, Austria). The sample size calculation was performed with the objective of demonstrating the non-inferiority of the 8-week treatment with EHO-85 compared to the control. The probable superiority of EHO-85 hydrogel at WAR was prefixed at 5%, with a standard deviation of 35%, as previously published in similar trials found in the literature. Therefore, a prespecified non-inferiority margin of 10% was established, as the minimum clinically relevant difference. Under the assumption of normality and taking into account a one-sided α-significance level equal to 0.025, 174 patients were considered necessary to reach a power of 80%. Estimating the rate of dropouts and withdrawals at 15%, a sample of at least 200 patients was estimated (100 in each group) would be necessary. Of these, patients with hard-to-heal ulcers were segregated for the study [18].

All analyses were performed on an ITT population. The comparison of the baseline between the two groups was performed by means of adapted tests: Student’s *t*-test, Mann-Whitney’s nonparametric test and chi-squared (χ^2^) test, according to the distribution and the characteristics of the variables Because the Shapiro-Wilk’s test showed that quantitative variables did not have a normal distribution, the non-parametric Wilcoxon-Mann-Whitney’s test was used to evaluate the uniformity of baseline variables in intervention and control groups. Moreover, homogeneity of variances in the two groups was assessed using the Levene’s test (Brown-Forsythe’s test in highly skewed distributions). This was due to the fact that the analysis of medians is more robust compared to the means. In the results, the medians and means of the study variables have also been shown, regardless of the results of the previous tests, to facilitate their evaluation. elative and absolute frequency tables were used for the description of the ordinal and qualitative variables. The determination of possible significant differences between the baseline variables of each group (intervention and control) was performed using the χ^2^ method.

### 4.6. Analyses of Efficacy Results

Statistical significance was determined using an alpha (α) level, or the probability of rejecting the null hypothesis when it is true. All hypothesis tests were set with α significance level of 0.05 (5%). The superiority of the study treatment was based on 95% confidence interval analyses of relative WAR. Considering the large deviation of the distributions of wound regression variables from normality, together with the difficulty of normalizing such distributions, non-parametric Mann-Whitney’s tests for the comparison of the quantitative variables of both groups were used. Comparisons of qualitative variables between groups were performed with the χ^2^ test or Fisher’s exact test, when the frequencies were less than 5 in the contingency tables. In addition, variables involving temporal evolution were evaluated by means of a Kaplan-Meier’s approach and followed by a Log-Rank’s curve comparison test. In addition, the Cox’s proportional hazards regression model was used to assess variations in WAR, taking into account thetime as a covariate. Scale variables were shown by their means ± SD or means and standard errors of the means, medians and ranges. Median differences were given with 95% CI. Nominal and ordinal variables were displayed by number of patients implicated and percentages.

## Figures and Tables

**Table 1:** Baseline characteristics of ITT patients with torpid ulcers of more than six months duration.

			EHO-85 (*n* = 35)		VariHesive (*n* = 34)		
Characteristic			Mean/No.	*SD/%*	Mean/No.	*SD/%*	*p*
Women			23	65.7%	25	73.5%	
Age (years)			74.06	14.86	79.03	14.80	
BMI (kg/m^2^)			27.04	6.85	29.61	9.91	
Diabetes mellitus			10	28.6%	9	26.5%	
Current smoker			4	11.4%	2	5.9%	
Alcohol (yes)			5	14.3%	1	2.9%	
Patient status							0.561
Health-center care	16	23.2%	10	28.6%	6	17.6%	
Home care	34	49.3%	16	45.7%	18	52.9%	
Residential care (institutionalized)	19	27.5%	9	25.7%	10	29.4%	
Degree of autonomy							0.389
Walks easily	14	20.3%	9	25.7%	5	14.7%	
Walks with difficulty	22	31.9%	9	25.7%	13	38.2%	
Confined to bed	33	47.8%	17	48.6%	16	47.1%	
Lower-limb mobility							0.274
Full mobility	17	24.6%	10	28.6%	7	20.6%	
Reduced mobility	24	34.8%	9	25.7%	15	44.1%	
Immobility	28	40.6%	16	45.7%	12	35.3%	
Blood test							
Serum albumin (3.40–5.00 g/dL)	3.61			0.48	3.57	0.51	0.665
Creatinine clearance (80–120 mL/min)	106.1			44.1	111.4	47.7	0.43

**Table 2:** Hard-to-heal ulcers.

	EHO-85 (*n* = 35)		VariHesive (*n* = 34)	
Ulcer	Mean/No.	*SD/%*	Mean/No.	*SD/%*
Etiology				
Venous	14	40.0%	14	41.2%
Ankle brachial index	0.99	0.09	1.04	0.18
Pressure	20	57.1%	19	55.9%
EPUAP II	7	35.0%	7	36.8%
EPUAP III	13	65.0%	12	63.2%
Diabetic foot	1	2.9%	1	2.9%
Wagner I	1	100.0%	0	0.0%
Wagner II	0	0.0%	1	100.0%
Ulcers/patient				
1	21	60.0%	18	52.9%
2	8	22.9%	10	29.4%
≥3	6	17.1%	6	17.6%
Evolution time (months)	15.74	9.72	17.75	9.46
Wound area (cm^2^)	6.67	11.04	4.65	4.75
Wound area				
≤10 cm^2^	23	65.7%	23	67.6%
>10 cm^2^	12	34.3%	11	32.4%
Granulation tissue (% over total ulcer)	70.57	41.40	83.13	31.79
Exudate				
None	5	14.3%	4	11.8%
Low	17	48.6%	13	38.2%
Intermediate	10	28.5%	14	41.2%
High	3	8.6%	3	8.8%
Recurrent ulcer	20	57.1%	16	47.1%
Previous hospitalization(s) due to treated ulcer(s)	8	22.9%	2	5.9%

## Data Availability

The data presented in this study are available on request from the corresponding author. The data are not publicly available due to the privacy of the patients who assisted in the research.

## Supplementary Materials

The following supporting information can be downloaded at: https://www.mdpi.com/article/10.3390/gels9120962/s1, Table S1: Collaborating nurses (sub-investigators) by center; Table S2: Exclusion criteria; Table S3: Specific exclusion criteria for each type of ulcer.
