# Supplementary material for: Hard-to-Heal Wound Healing: Superiority of Hydrogel EHO-85 (Containing Olea europaea Leaf Extract) vs. a Standard Hydrogel. A Randomized Controlled Trial
Source: Gels. 2023 Dec 8;9(12):962. doi: 10.3390/gels9120962 (PMC10742797; doi:10.3390/gels9120962)
Supplement: Supplementary file 1 [file gels-09-00962-s001.zip › Table-S1.pdf]

Table S1. Collaborating nurses (sub-investigators) by center

Clinical-trial center and principal investigator	Researcher nurse	Health center and nursing center
**Centro Salud Aeropuerto**	Alberto Garrido Arroyo	Centro Salud Aeropuerto
Concepción Mansilla Pedregosa (PI)	Mercedes Garrido Aranda	Centro Salud Aeropuerto
	María José Ibáñez Fernández	Centro Salud Aeropuerto
	María José Millán Ayala	Centro Salud Aeropuerto
	Pilar Lucena Díaz	Centro Salud Aeropuerto
	Dolores Lozano Mesas	Centro Salud Aeropuerto
	Rosa Mª Coronado Molina	Centro Salud Aeropuerto
	Rosa Mª Martínez Guillén	Centro Salud Aeropuerto
	Aileen Mª Serrano Ramón	Centro Salud Aeropuerto
	Gema Mª Delis Carrión	Centro Salud Aeropuerto
	Aurora Alameda López	Centro Salud Aeropuerto
**Centro Salud Castilla del Pino**	Amelia Sanjuan Espiñeira	Centro Salud Castilla del Pino
Raquel Mª López López (PI)	Eulalia Navarro Juan	Centro Salud Castilla del Pino
	Fernanda Casado Salinas	Centro Salud Castilla del Pino
	María Angeles Reinoso Araque	Centro Salud Castilla del Pino
	Alejandro Contreras Beato	Centro Salud Castilla del Pino
	Francisco Viana Miranda	Centro Salud Castilla del Pino
	Exiquio Murillo Sánchez	Centro Salud Castilla del Pino
	Isabel María Luque Huertas	Centro Salud Castilla del Pino
	Mónica Merlo Viso	Centro Salud Castilla del Pino
	Pedro Andrés Galey Chica	Consultorio La Marina - Figueroa
	Francisco José Vidal Maestre	Residencia Vitalia - San Rafael
	Natalia Acosta Ceballos	Residencia Vitalia - San Rafael
	Marta Izquierdo Prados	Residencia Vitalia - San Rafael
	María Reyes Bravo	Residencia Vitalia - San Rafael
	Carmen Burg Gómez de Mercado	Residencia FEPAMIC
	María Inmaculada González Muñoz	Residencia FEPAMIC
	Ana María Pérez de la Lastra Zamorano	Residencia FEPAMIC
	Tamara Albañil Frías	Residencia FEPAMIC
	Ana María Crespo Clavellina	Residencia FEPAMIC
	Dr. Jose María Jiménez Páez	Residencia Figueroa
	María Dolores Salamanca Bautista	Residencia Figueroa
**Centro Salud Centro de Córdoba**	Lourdes Garcia Vázquez	Centro Salud Centro de Córdoba
Juan Antonio Rodríguez Salamanca (PI)	Gema Ordóñez Romero	Centro Salud Centro de Córdoba
	Juana Valle Campos	Centro Salud Centro de Córdoba
	Gloria Martinez Galera	Centro Salud Centro de Córdoba
	Inmaculada Ruiz Prieto	Centro Salud Centro de Córdoba
	Cristina Gil Muñoz	Centro Salud Centro de Córdoba
	Inmaculada Garcia Gómez	Centro Salud Centro de Córdoba
	Estefania Montiel Garcia	Centro Salud Centro de Córdoba
	Inmaculada López Barranco	Centro Salud Centro de Córdoba
	Cristina Varo Cadenas	Residencia Virgen de los Dolores
**Centro Salud Fuensanta**	Amalia Pastrana Sánchez Crespo	Centro Salud Fuensanta
Antonia Dominguez Ramírez (PI)	María Jose Muñóz Urbano	Centro Salud Fuensanta
	Inmaculada Algar Algar	Centro Salud Fuensanta
	María Elena Fernández Díaz	Centro Salud Fuensanta
	Nieves Díaz Sedano	Centro Salud Fuensanta
	Jose Fernando Cejas Delgado	Centro Salud Fuensanta
	Elena Ponferrada León	Centro Salud Fuensanta
	María Jesus Jiménez Canales	Centro Salud Fuensanta
	Carmen Sánchez Pérez	Consultorio Los Angeles (Alcolea)
	Jesús Vicente Murcia Martínez	Residencia El Yate (Alcolea)
**Centro Salud Guadalquivir**	Maria del Carmen Heredia Lozano	Centro Salud Guadalquivir
Feliciano Santos Blanco (PI)	Carmen Miras García	Centro Salud Guadalquivir
	Yolanda Sánchez Palomo	Centro Salud Guadalquivir
	Gloria Navarro Luque	Centro Salud Guadalquivir
	Carmen Márquez Córdoba	Centro Salud Guadalquivir
	Rosa María Troyano Pérez	Centro Salud Guadalquivir

Clinical Trial Centers and Principal Investigator	Nurse researchers	Health-center and nursing-centers
**Centro Salud Huerta de la Reina**	Jose Juan Garés Laguna	Centro Salud Huerta de la Reina
Francisca Cuevas Pareja (PI)	Adoración Muñoz Alonso	Centro Salud Huerta de la Reina
	Fernanda Moreno Vargas	Centro Salud Huerta de la Reina
	Ana Belén Castellano Cano	Centro Salud Huerta de la Reina
	María Antonia Salcines Muñoz	Centro Salud Huerta de la Reina
	Francisco Escribano Villanueva	Centro Salud Huerta de la Reina
	Francisca Tocado Narganes	Centro Salud Huerta de la Reina
**Centro Salud Levante Sur**	*Juana Pérez Valero*	Centro Salud Levante Sur
María Muro Guerrero (PI)	*Teresa Rubio Berlanga*	Centro Salud Levante Sur
	Margarita Madrid Querol	Centro Salud Levante Sur
	Eva Maria Luque Marin	Centro Salud Levante Sur
	Francisco Manuel Jurado Rojo	Centro Salud Levante Sur
	Rafaela Simoni Pedrera	Centro Salud Levante Sur
	Matilde Membrillo Fuentes	Centro Salud Levante Sur
	Antonio García Ocaña	Centro Salud Levante Sur
	Francisco López Torres	Centro Salud Levante Sur
	Montserrat Encuentra Lerma	Centro Salud Levante Sur
	Lidia Gutiérrez Sánchez	Centro Salud Levante Sur
	Aurora Servanda Martínez Pérez	Centro Salud Levante Sur
	Brígida Jurado Galván	Centro Salud Levante Sur
**Centro Salud Levante Norte**	Carmen Canales Salguero	Centro Salud Levante-Norte
Antonia Carmona Priego (PI)	Manuel Toledano Estepa	Centro Salud Levante-Norte
	Carmen María Fuentes Madrid	Centro Salud Levante-Norte
	Antonio Emilio Martínez Más	Centro Salud Levante-Norte
	Pilar Ortiz Morales	Centro Salud Levante-Norte
	Maria Dolores Rubio González	Centro Salud Levante-Norte
	Sacramento Rosel Castro	Centro Salud Levante-Norte
	Carmen María Rodríguez Garriguet	Centro Salud Levante-Norte
	Carmen Albañir Albalá	Centro Salud Levante-Norte
	Belén Jiménez Holgado	Centro Salud Levante-Norte
**Centro Salud Lucano**	Mª Angeles Rodríguez Castillo	Centro Salud Lucano
Antonio González Delgado (PI)	Antonia Becerra Fernandez	Centro Salud Lucano
	Jose Manuel Fernández Granados	Centro Salud Lucano
	Manuela Hidalgo Morillo	Centro Salud Lucano
	Antonio Jesús Cecilla Moral	Residencia Santísima Trinidad
	Matilde Cano Merlo	Residencia Santísima Trinidad
**Centro Salud Occidente**	Manuela Urbano Priego	Centro Salud Occidente
Caridad Dios Guerra (C)	María Dolores López Espejo	Centro Salud Occidente
	Rosalía Serrano Berni	Centro Salud Occidente
	María Azahara García Bono	Centro Salud Occidente
	Carmen Fernández Gutierrez	Consultorio El Higuerón
	María Salud Nieto González	Consultorio El Higuerón
	Rosario Dios Guerra	Consultorio Villarrubia
**Centro Salud Poniente**	Magdalena García Carrasco	Centro Salud Poniente
Mª Dolores Marín Alfaro (PI)	Manuel Moreno Rodríguez	Centro Salud Poniente
	Inmaculada Guzmán Castilla	Centro Salud Poniente
	Araceli Alcaide Guirao	Centro Salud Poniente
	Teresa Martinez de la Torre	Centro Salud Poniente
	Luis Heredia Borrego	Centro Salud Poniente
	Rosell de la Oliva Ramírez	Centro Salud Poniente
	Sergio Garrido Bollo	Centro Salud Poniente
	Jorge Rafael Padilla Maestre	Centro Salud Poniente
	Palmira I. Gallego Huertas	Centro Salud Poniente
**Centro Salud Santa Rosa**	Angela María Gonzaléz García	Centro Salud Santa Rosa
Santiago Cruz Velarde (PI)	Federico Urbano Ramirez	Centro Salud Santa Rosa
	Milagrosa Aguilar Villalba	Centro Salud Santa Rosa
	Mª Carmen Luna Poyato	Centro Salud Santa Rosa
	Sonia Calero Juárez	Centro Salud Santa Rosa
	Cristina López Olivares	Residencia ORPEA Centro
	Ana Pozo Olivares	Residencia ORPEA Centro
	Juan Jose García Zamudio	Residencia ORPEA Centro
	Teresa Ruiz López	Residencia ORPEA Sierra
	Mª Encarnación Pulido Sanchez	Consultorio Bda. El Naranjo

Clinical Trial Centers and Principal Investigator	Nurse researchers	Health-center and nursing-centers
**Centro Salud Sector Sur (Santa Victoria)**	Rosalía Espino Navarro	Centro Salud Sector Sur (Santa Victoria)
José Tomás Linares García (PI)	Ezequiel Jiménez Priego	Centro Salud Sector Sur (Santa Victoria)
	Jose Antonio Santaella Alcaide	Centro Salud Sector Sur (Santa Victoria)
	Inés Calvo Cabrera	Centro Salud Sector Sur (Santa Victoria)
	Raquel López Valero	Centro Salud Sector Sur (Santa Victoria)
	Ana Morgado Ramírez	Centro Salud Sector Sur (Santa Victoria)
**Centro Salud Bujalance**	Antonia Mohedo Caballero	Centro Salud Bujalance
Esteban Luis García Lara (PI)	Catalina Gómez Diaz	Centro Salud Bujalance
	José Ramón Serrano González	Centro Salud Bujalance
	Antonio Luna Mantas	Consultorio Cañete de las Torres
	Juan Antonio Quiros Blázquez	Consultorio El Carpio
	Carmen López Jimenez	Consultorio Villafranca de Córdoba
**Centro Salud Montoro**	Inmaculada Vega-Leal Bellido	Centro Salud Montoro
Beatriz Alcalá Aguilera (PI)	María José Luna Romero	Centro Salud Montoro
	Araceli Carretero Gómez	Centro Salud Montoro
	Amelia Camacho Buenosvinos	Centro Salud Montoro
	Virgina González Pérez	Centro Salud Montoro
	María del Mar Maya Cabrera	Residencia Jesús Nazareno
	María Dolores Baeza Cerro	Residencia Jesús Nazareno
	Isabel Sánchez Gálvez	Residencia Jesús Nazareno
	Pilar Mayorga Hortelano	Residencia Jesús Nazareno
	Joaquín Ruz Ramírez	Consultorio Adamuz
	María del Pilar Jurado Rueda	Consultorio Pedro Abad
	Mª Ángeles Delgado Uceda	Consultorio Villa del Río
	Cristina Gracia Rivera	Consultorio Villa del Río
	Dolores Ramirez Carmona	Consultorio Villa del Río
**Centro Salud Fuente Palmera**	Jesús Poyato Velasco	Centro Salud Fuente Palmera
Marcial Caballero Arroyo (PI)	Maria del Valle Pavón Santacruz	Centro Salud Fuente Palmera
**Centro Salud La Carlota**	Antonia Cuesta Plata	Centro Salud La Carlota
Aranzazu Sempere Gracia (PI)	Enrique de la Cueva Montesinos	Centro Salud La Carlota
	Rafael Campos López	Centro Salud La Carlota
	Asunción Parias Salas	Consultorio La Victoria
**Centro Salud Palma del Río**	Margarita Aguilar Berastegui	Centro Salud Palma del Río
Dolores Lopera Marín (PI)	MªAngeles Jimenez Sanchez	Centro Salud Palma del Río
	Rafael Molero De la Mata	Centro Salud Palma del Río
	Carlos Enrique Cabello Jaime	Centro Salud Palma del Río
	Eulalia Prieto Vilela	Centro Salud Palma del Río
	Alonso Guerra Milla	Centro Salud Palma del Río
**Centro Salud Posadas**	Ana María Molina Moreno	Centro Salud Posadas
Antonia Quero Vilchez (PI)	Begoña González Vallin	Centro Salud Posadas
	Eva Dorado Espinosa	Centro Salud Posadas
	Victoria Migallón Sanchez	Centro Salud Posadas
	Francisco Sánchez Guerrero	Centro Salud Posadas
	Elena Gómez Moreno	Centro Salud Posadas
	Raquel Bravo Martínez	Consultorio Hornachuelos
	Isabel Luque Cantarero	Residencia San Bernardo
	Mónica López Muriel	Consultorio Los Mochos
**Centro Salud Montilla**	Eva María Romero Bonilla	Centro Salud Montilla
María Dolores de la Cruz Hidalgo (PI)	Juan Manuel Vilas Casado	Centro Salud Montilla
	María Dolores Vilchez Gallegos	Centro Salud Montilla
	María Luisa Clavero Berral	Centro Salud Montilla
	Antonio Rodríguez Estepa	Centro Salud Montilla
	Gonzalo Garramioza Robles	Centro Salud Montilla
	Josefa Márquez Martínez	Centro Salud Montilla
	Rosa María Salido Bellido	Centro Salud Montilla
	Teresa Luque Llamas	Centro Salud Montilla
	Isabel Mengual García	Centro Salud Montilla
	Manuel Luque-Romero Sánchez	Centro Salud Montilla
	Encarnación Luque Reyes	Centro Salud Montilla
	María Felisa Luque Martínez	Centro Salud Montilla
	Ana Isabel Calero García	Centro Salud Montilla

Clinical Trial Centers and Principal Investigator	Nurse researchers	Health-center and nursing-centers
**Centro Salud Montilla (cont.)**	María José Pérez Pérez	Centro Salud Montilla
María Dolores de la Cruz Hidalgo (PI)	Marcos Bellido Sánchez	Residencia San Juan de Dios
	Verónica Galán Marín	Residencia San Juan de Dios
	Carmen María Comino Montilla	Residencia San Juan de Dios
**Centro Salud Lucena I y II**	Nicolasa García Gallardo	Centro Salud Lucena
Antonio José Rivas Ogalla (PI)	María Isabel Luna Corredera	Centro Salud Lucena
	Inmaculada Cañete Muñoz	Centro Salud Lucena
	María Luisa González Delgado	Centro Salud Lucena
	Inmaculada Jiménez Corredera	Centro Salud Lucena
	Juan B. Guerrero Muñoz	Centro Salud Lucena
	Antonio Martos Cárdenas	Centro Salud Lucena
**Centro Salud Cabra**	Purificación María Servian Rodríguez	Centro Salud Cabra
Matilde Romero López (PI)	Leonardo Llado Salas	Centro Salud Cabra
	Gertrudis Roldán Molina	Centro Salud Cabra
	María Pilar Esteo Domínguez	Centro Salud Cabra
	María del Rosario Altés Comino	Centro Salud Cabra
	María Teresa Aguilar del Río	Centro Salud Cabra
	José Manuel Chacón Jiménez	Centro Salud Cabra
	María Ruz Ruiz	Centro Salud Cabra
	María del Mar Moreno Ruiz	Centro Salud Cabra
	Juan Rabadán López	Centro Salud Cabra
	Ana Ortiz Roldán	Residencia PROMI
	Mª Sierra Pérez Gallego	Residencia PROMI
	María José Cubero Muñoz	Consultorio Doña Mencía
	Angeles Lopera Parraga	Consultorio Nueva Carteya
	Alba María Pérez-Vico Contreras	Consultorio Nueva Carteya
**Centro Salud Fernan Nuñez**	Miguel García Jiménez	Centro Salud Fernan Nuñez
Isabel Alcaide Aguilar (PI)	Ana Arjona Martín	Centro Salud Fernan Nuñez
	María Angeles Carmona López	Centro Salud Fernan Nuñez
	María Jesús Pérez Cobos	Centro Salud Fernan Nuñez
