# Supplementary material for: Hard-to-Heal Wound Healing: Superiority of Hydrogel EHO-85 (Containing Olea europaea Leaf Extract) vs. a Standard Hydrogel. A Randomized Controlled Trial
Source: Gels. 2023 Dec 8;9(12):962. doi: 10.3390/gels9120962 (PMC10742797; doi:10.3390/gels9120962)
Supplement: Supplementary file 1 [file gels-09-00962-s001.zip › Table-S2.pdf]

Table S2. Exclusion criteria

Diabetes mellitus with HbA1c >9.5%
Serum albumin <2.5 g/dl
Severe renal insufficiency
Hepatic insufficiency
Connective-tissue disease
Systemic infection
Local-wound infection
Pregnancy or lactation
Systemic-corticosteroid therapy
Treatment with immunosuppressants
Tumor-necrosis factor (TNF) inhibitor therapy
PPAR-gamma agonist treatment
In parallel, VLU and DFU had to have posterior tibial pulse and/or pedal present, and the arm-to-ankle pressure index (ABPI) had to be ≥0.8
