# Supplementary material for: Hard-to-Heal Wound Healing: Superiority of Hydrogel EHO-85 (Containing Olea europaea Leaf Extract) vs. a Standard Hydrogel. A Randomized Controlled Trial
Source: Gels. 2023 Dec 8;9(12):962. doi: 10.3390/gels9120962 (PMC10742797; doi:10.3390/gels9120962)
Supplement: Supplementary file 1 [file gels-09-00962-s001.zip › Table-S3.pdf]

Table S3. Specific exclusion criteria for each type of ulcer

**Pressure-ulcer patients**
Ulcers whose main cause is urinary or fecal incontinence.
Without willingness and/or availability of caregivers to follow care guidelines during the study.
**Venous leg-ulcer patients**
History of venous surgery in previous two months.
Episodes of venous thrombosis in previous three months.
History of intermittent claudication in previous three months.
Absence of posterior tibial and/or pedal pulse in extremity where ulcer was located.
Ankle-brachial index (ABI) <0.8 in previous six months.
Patient and/or caregivers unwilling and/or unavailable to allow daily use of compression bandage for the duration of the study.
**Diabetic- foot patients**
Not diagnosed with type 1 or 2 diabetes mellitus.
Ulcer with Charcot arthropathy (neuropathic arthropathy).
Absence of posterior tibial and/or pedal pulse in extremity where ulcer was located.
Ankle-brachial index (ABI) <0.8 in previous six months.
